# Real-world outcomes of early and deferred anti-VEGF treatment in diabetic macular oedema in patients with type 1 diabetes

**DOI:** 10.1080/07853890.2025.2530218

**Published:** 2025-07-09

**Authors:** Joonas Wirkkala, Anna-Maria Kubin, Pasi Ohtonen, Nina Hautala

**Affiliations:** a Department of Ophthalmology, Oulu University Hospital, Oulu, Finland; b Research Unit of Clinical Medicine, University of Oulu, Oulu, Finland; c Medical Research Center, University of Oulu, Oulu, Finland; d Research Service Unit, Oulu University Hospital, Oulu, Finland; e Research Unit of Surgery, Anesthesiology and Intensive Care, University of Oulu, Oulu, Finland

**Keywords:** Type 1 diabetes, diabetic retinopathy, diabetic macular oedema, intravitreal anti-VEGF

## Abstract

**Background:**

Intravitreal anti-vascular-endothelial growth factor (anti-VEGF) injections have revolutionized the treatment of diabetic macular oedema (DME). The effect of early initiation of anti-VEGF treatment with good baseline visual acuity on treatment outcomes was compared with that of deferred treatment in patients with type 1 diabetes (T1D) and DME.

**Patients and methods:**

This was a population-based real-world retrospective cohort study of anti-VEGF-treated T1D patients with DME between 2010–2020. The data included age, sex, diagnosis of T1D and DME, severity of retinopathy, vision, duration of DME, and number of injections.

**Results:**

In total, 266 anti-VEGF-treated DME episodes were included. The average age at the time of T1D diagnosis was 25士17years. At the onset of DME, the patients were 50士15 years old, the duration of T1D was 25士13 years, and 68% had non-proliferative and 32% had proliferative diabetic retinopathy. An average of 5.8 (SD 4.9) and 6.6 (SD 4.9) injections were required in the early treatment (baseline BCVA ≥75) and deferred treatment (<75 ETDRS letters) groups, respectively (*p* = 0.207). The DME duration was similar in both groups ((10.4 (SD 8.8) vs. 10.7 (SD 8.8) months, *p* = 0.81)). In 71% of the cases, no recurrence of DME was noted for at least two years after the resolution of DME. After treatment, visual acuity was 5.4 (95% CI 3.4 to 7.5) ETDRS letters higher in the early vs. deferred treatment groups, respectively (*p* < 0.001).

**Conclusion:**

Early treatment of DME with higher baseline BCVA may result in better visual outcomes in T1D patients, and delayed treatment may decrease the possibility of recovery of visual acuity. The duration of DME and number of injections were similar in the early and deferred treatment groups. Efforts to maintain proper visual function in T1D patients with DME are beneficial.

## THE KEY MESSAGES

**What is already known on this topic:**

The availability of intravitreal anti-VEGF agents has revolutionized the treatment and visual prognosis of DME. However, the most beneficial time to start treatment in patients with T1D and DME is unknown.

**What this study adds:**

Early treatment of DME with higher baseline BCVA seems to result in better visual outcomes in T1D patients.

**How this study might affect research, practice or policy:**

Work-aged individuals with T1D benefit the most from early scheduled anti-VEGF treatment of DME, and the delay in treatment may decrease the possibility of recovery of visual acuity.

## Introduction

Intravitreal anti-VEGF agents have revolutionized the treatment of diabetic macular oedema (DME) over the last two decades and are currently the first-line treatment for DME [1]. Frequent and continuous treatment creates an increasing burden for healthcare organizations in industrialized countries, since the number of individuals with diabetes is expected to increase from 537 million in 2021 to 783 million by 2045 [2]. The modern treatment of DME and proliferative diabetic retinopathy (PDR) with anti-VEGF agents has significantly reduced the rate of visual impairment due to diabetic retinopathy (DR) and its complications [1,3,4].

Despite convincing evidence regarding the efficacy of anti-VEGF treatment in DME during the last decade, only a limited number of studies have reported DME treatment outcomes in patients with type 1 diabetes (T1D) [5]. In particular, it has been established that the clinical features and risk factors of DME might differ between patients with T1D and type 2 diabetes (T2D) [6,7]. The most beneficial time for the initiation of anti-VEGF treatment in working-age patients with T1D and DME is also not known.

This study was designed to assess the visual outcomes of T1D patients with DME in a real-life setting. Our aims included the impact of early versus deferred initiation of anti-VEGF therapy on primary outcomes: the likelihood of DME recurrence following the initial episode and changes in best-corrected visual acuity (BCVA) from the initial treatment to the conclusion of the follow-up period. Secondary outcomes included overall treatment burden reflected in the number of required intravitreal injections and the duration of DME episodes. This comparative analysis provides real-world results about the timing of intervention with anti-VEGF agents and its implications for long-term visual outcomes in patients with T1D.

## Patients and methods

This study was conducted at the Oulu University Hospital. This study followed the tenets of the Declaration of Helsinki of the World Medical Association. The Oulu University Hospital Research Committee was informed and deemed that no official ethical approval was required. This was a retrospective, register-based study, and informed consent was not obtained from the participants. Complete anonymity was ensured, and the article did not include any data that could identify the person.

This population-based, real-life cohort study was performed on all adult patients with T1D who presented at Oulu University Hospital with DME between December 1, 2010, and December 31, 2020. The hospital’s electronic patient database was used to identify patients using the ICD-10 (International Classification of Diseases) diagnosis codes for diabetic maculopathy (H36.1) and type 1 diabetes (E10.3). Clinically relevant macular oedema was considered present if optical coherence tomography (OCT) showed intra- or subretinal fluid in the macula with a central retinal thickness of ≥300 μm. Demographic data included parameters for age, sex, age at the time of diabetes onset, duration of diabetes, severity of DR at the time of DME onset, laterality of DME, treatment (laser treatment, intravitreal treatments, combination of the two), and time of follow-up. The current protocols for DME treatment were adhered to [1,8]. In Finland, bevacizumab is commonly used as the first-line intravitreal drug for DME. Anti-VEGF treatment is usually initiated with 3 monthly injections and then, if needed, continued as treat-and-extend- or pro re nata (PRN) regimens. Patients with extrafoveal macular oedema located ≥500 μm from the central fovea were either observed or subjected to macular laser therapy depending on the location and amount of intraretinal or subretinal fluid. Patients with central macular oedema within 500 μm of the fovea were primarily subjected to intravitreal anti-VEGF treatment. Patients were subjected to combination treatment with anti-VEGF and macular laser in cases of subsequent central and extrafoveal oedema. Early treatment with anti-VEGF agents was initiated at the time of DME diagnosis with no markedly declined vision. The treatment of DME was considered deferred in patients with a decrease in visual acuity to less than 75 ETDRS letters before the initiation of anti-VEGF treatment after DME was first observed according to the assessment of the treating physician. A single DME episode was defined as the period from the diagnosis of DME to the resolution of the oedema, indicated by a central retinal thickness of less than 300 μm, although some residual fluid might be present. After achieving the resolution of DME following anti-VEGF-treatment, subsequent episodes of DME were considered as part of the initial episode if recurrence occurred within 4 months. If recurrence occurred after 4 months, it was considered a new episode of DME. The BCVA was documented as ETDRS-letters during DME treatment and follow-up. The severity of DR was based on the 5-scale classification system of the Finnish Current Care Guidelines for Diabetic Retinopathy [8]. Co-existing active proliferative diabetic retinopathy (PDR) was an exclusion criterion.

Statistical analyses were performed using the SPSS for Windows (IBM Corp. Released 2019. IBM SPSS Statistics for Windows, Version 26.0. Armonk, NY: IBM Corp) and SAS (version 9.4 SAS Institute Inc., Cary, NC, USA) Continuous variables were presented as means with standard deviation (SD) and minimum and maximum values unless otherwise stated. Categorical variables are presented as frequencies (percentages). The independent samples t-test was used for intergroup comparisons of patient-related data. A linear mixed model (LMM) was used for the primary outcome comparison. In the LMM, patients and eyes were set as random effects to handle within-patient and eye correlations. The Kaplan-Meier survival curve was created, and a multivariable-adjusted Cox frailty model was used to determine if there were differences in the timing and proportion of eyes with recurrent DME based on the baseline visual acuity. The frailty model was adjusted for age, sex, duration, and severity of DR, and the patient was used as the frailty factor. A 95% confidence interval (95% CI) was presented with the results of the LMM and frailty models. A two-sided p value <0.05 was considered statistically significant.

## Results

A total of 266 anti-VEGF-treated DME episodes in 153 eyes of 108 patients with T1D and DME were included in this study. 59 (55%) patients were male (Table 1). Of 266 DME episodes, 144 (54%) were anti-VEGF-treatment-naive. Among patients with BCVA ≥75 (early treatment) and <75 ETDRS letters (deferred treatment), 10 (23%) and 14 (22%) had history of focal macular laser. All 35 patients with PDR had received prior panretinal photocoagulation. The average age at T1D diagnosis was 25士17 years. DR was diagnosed 17士11 years later, on average. The mean onset of DME occurred 9士7 years after the onset of DR, and the time interval from the diagnosis of T1D to that of DME was 25士13 years. The average age at the time of DME diagnosis was 50士15 (Table 1). At the time of DME onset, 73 (68%) patients had non-proliferative DR and 35 (32%) patients had inactive PDR. No DME recurrence during the follow-up period was noted in 71% of the cases.

**Table 1.** Baseline characteristics of the study participants

	BCVA < 75 ETDRS letters *N* = 65	BCVA ≥ 75 ETDRS letters *N* = 43	p^†^
Patients, n (%)			
Males	34 (52)	25 (58)	0.35
Eyes	94 (72)	59 (67)	0.29
DR class when DME was diagnosed first time, n (%)			0.36
NPDR	41 (63)	32 (74)	
PDR	24 (37)	11 (26)	
Previous focal macular laser treatment	14 (22)	10 (23)	0.59
Age at DM, years, mean (SD) [min-max]	26 (17) [2-67]	23 (17) [1-59]	0.37
Age at DME, years, mean (SD) [min-max]	52 (15) [24-79]	48 (15) [18-76]	0.17
Time From T1D to DR, years, mean (SD) [min-max]	17 (11) [0-52]	17 (12) [0-52]	0.74
Time From T1D to DME, years, mean (SD) [min-max]	26 (13) [0-58]	25 (14) [0-57]	0.61

BCVA = best-corrected visual acuity, DME = diabetic macular edema, DR = diabetic retinopathy, ETDRS = early treatment diabetic retinopathy study, NPDR = non-PDR, PDR = proliferative diabetic retinopathy, SD = standard deviation, T1D = type 1 diabetes

^†^statistical significance at p < 0.05

**Figure 1.**
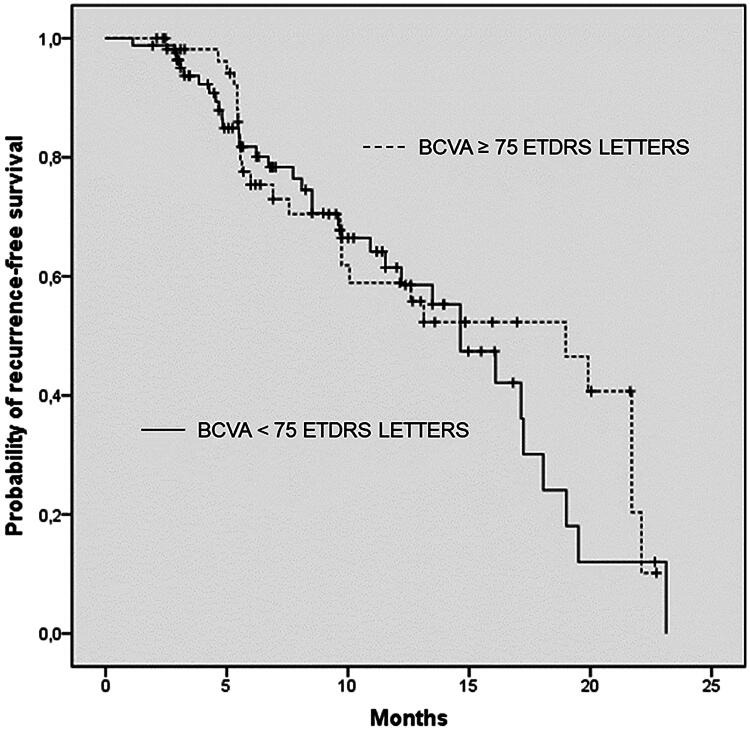
Probability of the recurrent DME during the 24 month-period.

According to the multivariable adjusted Cox frailty model, patients with BCVA <75 letters before receiving their first treatment exhibited a slight increase in the risk of needing a new treatment period and there was a 2% increase in risk compared to other patients. However, this increase was not statistically significant (hazard ratio = 1.02, 95% CI 0.65 to 1.62, *p* > 0.90) (Figure 1).

At the end of the treatment, visual acuity was 5.4 (95% CI 23.4 to 7.5, *p* < 0.001) ETDRS letters higher in the early treatment group than in the deferred treatment group. At the end of study, 9/59 (15%) and 54/94 (57%) eyes gained at least one ETDRS line in the early vs. deferred treatment groups, respectively. After treatment of a DME episode, in the deferred treatment group, 10/101 (9.9%) eyes achieved a BCVA of 75 or better ETDRS letters.

**Figure 2.**
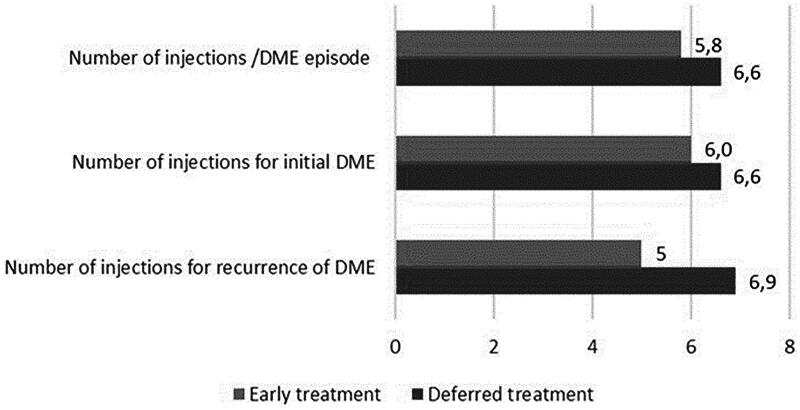
The number of anti-VEGF injections required for treatment of DME.

An average of 5.8 (SD 4.9) and 6.6 (SD 4.9) injections were required for one episode of DME in the groups with BCVA ≥75 (early treatment) and <75 ETDRS letters (deferred treatment) at baseline, respectively (difference 0.84, 95% CI −0.5 to 2.2, *p* = 0.21). The initial episode of DME required 6.0土4.6 and 6.6土4.7 anti-VEGF injections and the recurrence of DME 5.0土3.0 and 6.9土5.7 injections in these groups, respectively (Figure 2). Over half (52%) of the cases were treated with four injections and 65% with six injections. Twelve patients had a switch from bevacizumab to aflibercept during DME treatment. A total number of injections is shown in Table 2.

**Table 2.** Clinical outcomes of DME in patients with T1D.

	BCVA < 75 ETDRS letters *N* = 101	BCVA ≥ 75 ETDRS letters *N* = 165
BCVA (ETDRS letters), mean (SD) [min-max]		
At the onset of DME	66.3 (10.0) [35-74]	83.3 (3.9) [77-89]
At the end of study	74.5 (11.3) [35-92]	84.0 (5.0) [65-95]
Number of anti-VEGF-injections, mean (SD) [min-max]	9.1 (7.6) [1-48]	8.9 (9.2) [1-52]
Switch to aflibercept during DME treatment, n (%)	8 (7.9)	4 (2.4)

BCVA = best-corrected visual acuity, DME = diabetic macular edema.

**Figure 3.**
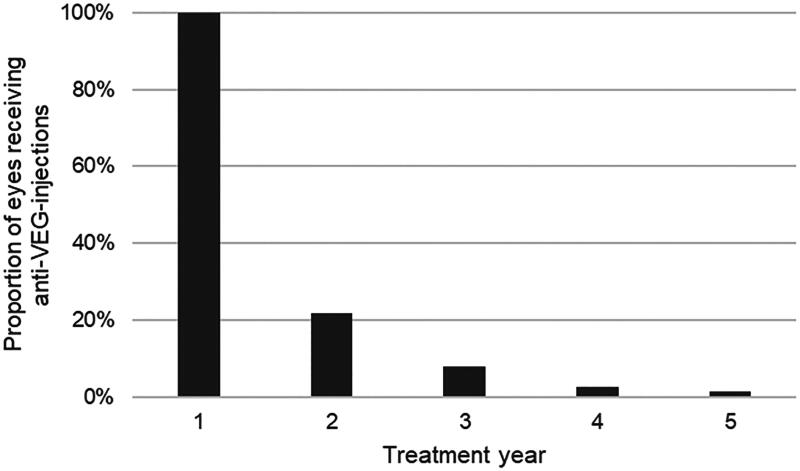
Proportion of eyes requiring anti-VEGF-injections during the 5-year follow-up.

In 71% of the cases, anti-VEGF injections were administered only during the first year of treatment, in 22% of the cases for two years and only in 8%, 3%, and 1% of the cases, patients needed anti-VEGF injections during the third, fourth, or fifth year after the onset of DME, respectively (Figure 3). In the first year of DME treatment, patients received an average of 4.8 injections, 3.0 injections during the second year, when needed, 2.5, 2.0 and 2.2 injections, respectively, during the following three years.

The average duration of DME treatment was similar in both groups (10.4 months in early treatment vs. 10.7 months in deferred treatment groups, difference 0.3, 95% CI −2.1 to 2.7, *p* = 0.81). After a year, continuous anti-VEGF treatment was needed for prolonged DME in 24/165 (15%) and 35/101 (35%) episodes in the early vs. deferred treatment group (chi-square *p* = 0.7). Furthermore, there was a statistically significant difference in BCVA between the groups, sustained during the first (*p* < 0.001) and second (*p* = 0.013) years of treatment. The respective BCVA in ETDRS letters was 85.2 after the first year and 83.1 after the second year in the early treatment group. In the deferred group, the BCVA was 75.5 and 73.6 ETDRS letters after the first and second years, respectively.

## Discussion

The results of this real-life study suggest that early anti-VEGF treatment of DME with higher baseline BCVA indicates better visual outcomes in patients with T1D than in those with deferred treatment initiation. These results suggest that delayed treatment with DME may decrease the possibility of full visual acuity recovery. Treatment of DME has revolutionized owing to the availability of anti-VEGF therapy, leading to the reduction of visual impairment caused by DME or PDR [1,3,4,9]. Numerous studies have demonstrated the efficacy of intravitreal anti-VEGF agents in the treatment of DME and beneficial visual outcomes in patients with either T1D or T2D [1,10–13]. However, the clinical features and risk factors of DME vary among these patient groups [6,7]. Known risk factors for DME in T1D include duration of diabetes, age, and diastolic blood pressure [14]. In agreement, our previous real-life results indicated that the average age of the patients with T1D and DME was 47 years and that the average duration of diabetes was 24 years at the time of the onset of DME, suggesting the need for proper visual function for this working-age group of patients [5].

In our recent study, patients with T1D and DME receiving the most ETDRS letters were treated with anti-VEGF injections, and the best long-term visual outcomes were achieved by combination treatment with both anti-VEGF and macular laser [5]. Accordingly, satisfactory long-term visual outcomes in DME were achieved by early intensive treatment with anti-VEGF injections [15]. Even in cases of no BCVA improvement, anti-VEGF treatment may improve contrast sensitivity in patients with DME [16], thus providing the best long-term safety in maintaining good visual function. In the present study, the visual acuity was significantly, 5 ETDRS letters higher, in the early treatment group with good baseline visual acuity (BCVA ≥75 ETDRS letters) compared to the deferred treatment group (BCVA <75 ETDRS letters) at the end of the treatment. It is also notable that patients with better baseline BCVA have less potential for gaining vision than those with lower baseline vision.

In contrast to previous studies suggesting the observation of DME until worsening of BCVA, several studies have demonstrated a decrease in BCVA in patients with deferred treatment for DME [5,16–18]. This may be explained by the development of chronic DME during the observation period, which may harm retinal function and deteriorate the visual prognosis of these patients. In a *post hoc* secondary analysis of the DRCR.net Protocol V study, a baseline subretinal thickness of > 300 μm and advanced level of DR increased the need for anti-VEGF treatment for decreased vision in eyes with good BCVA at the onset of DME and with initial observation [18]. The severity of DR at the onset of DME is strongly associated with patients with T1D with a longer duration of diabetes compared to those with T2D. In a population-based cohort of patients with T1D and DME, 25% of patients had PDR [5]. However, most studies on DME have included populations consisting mainly of patients with T2D.

Several treatment strategies for DME may be warranted, depending on an individual’s specific circumstances, baseline vision, CRT, societal level, or cost of treatment. A previous study suggested that cost savings can be achieved when patients with DME and good visual acuity are either observed or treated with laser compared to intravitreal aflibercept [19]. However, off-label bevacizumab is widely used as a first-line treatment in several countries, including Finland, with much lower costs than other available anti-VEGF agents. Moreover, recent studies showed no evidence of a significant difference in visual outcomes over a 2-year period between aflibercept monotherapy and treatment with bevacizumab first, with a switch to aflibercept in the case of suboptimal response [20,21]. These results suggest substantial cost savings on a societal level without sacrificing visual acuity gains when patients with DME were treated with bevacizumab instead of aflibercept [21]. In the current real-life setting, 71% of patients with T1D and DME had only one DME episode during the 5-year follow-up. One-fifth of the cohort received anti-VEGF injections during the second year, and a minority (8%, 3%, and 1%) during the third, fourth, or fifth year after the onset of DME, respectively. In the first year of DME treatment, patients received an average of 4.8 injections, 3.0 injections during the second year, when needed, 2.5, 2.0 and 2.2 injections, respectively, during the following three years. In addition, early initiation of anti-VEGF treatment seemed to require fewer injections than deferred treatment in the current study cohort. These results indicate that the burden and costs of anti-VEGF agents, especially bevacizumab, in the treatment of DME are quite reasonable compared to those of permanent visual loss.

Our study has certain limitations. First, the retrospective nature of the study might be considered a limitation, although this study reported treatment outcomes in a real-life setting. Second, the underlying risk factors for DME have not been comprehensively studied, and complete data on blood glucose levels, cholesterol levels, and kidney function are lacking. Third, treatment decisions were made by treating physicians by appraisal of visual acuity, severity of overall DR, CRT, and other OCT findings, and assessments might have varied between several retina specialists, despite the treatment guidelines and established clinical practice. Also, the exact length of the observation period between diagnosis of DME and initiation of treatment after vision falling in the patients in the deferred treatment group is not comprehensively documented. The population-based study cohort and the long real-world follow-up might be considered the strengths of the current study. In addition, the inclusion of only T1D patients with DME in the present study can be considered a strength in contrast to most studies including patients with both T1D and T2D. The pathogenesis and risk factors of DME differ between these patient groups, and combining these results is a possible source of bias.

## Conclusions

In conclusion, our results suggest that early treatment of DME with higher baseline BCVA may indicate better visual outcomes in T1D patients, although the duration of DME and the number of injections were similar in the early and deferred treatment groups. The treatment of DME before the development of chronic DME and reduction of BCVA is likely to save costs and vision in working-age individuals with T1D.

## Data Availability

The data supporting the findings of this study are available from the corresponding author, NH, upon reasonable request.
